# Nurses’ trust in managers and its relationship with nurses’ performance behaviors: a descriptive- correlational study

**DOI:** 10.1186/s12912-021-00653-9

**Published:** 2021-07-27

**Authors:** M. Hadi-Moghaddam, M. Karimollahi, M. Aghamohammadi

**Affiliations:** Department of Nursing, School of Nursing and Midwifery, Ardabil University of Medical Sciences, Ardabil, Iran

**Keywords:** Nurse-head nurse trust, Nurse-supervisor trust, Nurse performance behavior, Iran

## Abstract

**Background:**

Organizational trust is one of the most important issues in human relations that its importance in organizations is well known. Effective communication and cooperation between individuals require trust. On the other hand, the quality of a nurse’s trust in his/ her manager affects the behavior and performance of the nurse. The purpose of this study was to determine nurses’trust in managers (head-nurses and supervisors) and its relationship with nurses’ performance behavior.

**Methods:**

This descriptive-correlational study was performed with the participation of 431 nurses working in educational centers of Ardabil, Iran. The sampling method was stratified randomly. Questionnaires of the McAllister Trust and Paterson Job Performance were used. Data analysiswas performed using descriptive statistics (mean, standard deviation, frequency) and Chi-square testin SPSS v.22.

**Results:**

The results showed that the majority of nurses trusted head-nurses (80.3%) and supervisors (61.9%). In addition, most nurses’ functional behavior (92.1%) was reported as excellent. There was no significant relationship between nurse trust in head-nurse and nurses’functional behavior (*P* = 0.58), while it was significant between nurse-supervisor trust and nurses’ functional behavior (*P* = 0.03).

**Conclusion:**

The results of this study showed a relationship between nurse-supervisor trust and nurses’ functional behavior. Therefore, it is recommended ways to improve the trust between the treatment team, especially among nurses and supervisors are considered as one of the factors influencing the nurses’ behavioral performance.

## Introduction

During the last two decades, the concept of trust has received special attention in the field of organizational research [1, 2] and numerous investigators have emphasized the importance of such a concept in an organizational structure [3]. In addition, it is because trust is a fundamental aspect of human interaction and its importance becomes especially evident in an organizational structure. The realization of cooperation and effective communication can only be achieved in the presence of trust among individuals [4]. Researchers believe that as long as organizations operate within the boundaries of social structures, the existence of trust is a prerequisite since it is a crucial factor in such structures [5].

Furthermore, the advent of globalization and its effects on work conditions, the shift from a traditional organization to hierarchical organization, the change from power-based control into self-control and self-management, communicational flexibility, and delegation of authority and decision-making process to all levels of the organization, has led to a rapid increase of importance of the concept of trust in the governmental organizations [6]. In the era of fast-changing and transient relationships, trust has become a pivotal issue for organizations since it entails interpretation and inference of individuals’ motivations, personality, and morality. That is why efforts are being made by the chain of command to obtain a better understanding of such a concept to establish more effective cooperation in the organization [7]. Trust is described as a belief in someone’s or something’s reliability [8]. Lee, et al. (2013) defines organizational trust as “the individuals’ expectations of the networks of organizational connections and behaviors” [9]. Trust is a multidimensional concept which incorporates a variety of backgrounds such as interpersonal trust, organizational trust, societal trust, workplace trust, and the trust between the subordinates and supervisors [10]. Lewis and Weigert (1985) define trust as a bi-factor concept which consists of cognitive and affective components. The foundation of a cognitive-based trust is cognitive reasoning. Contrarily, affective trust is built on emotional and social contracts that exceed the boundaries of professional or business relationships [11]. In an environment of trust, numerous positive organizational outcomes can be observed. On the contrary, there are consequences to the presence of workplace distrust which can namely be participation reluctance, low quality work, and the need to enforce control [12]. Trusting the supervisor means that the employees believe that their superiors have their best interest at heart [13]. Bell and Menguc (2002) defines trusting the supervisor as Organizational Citizenship Behaviors)OCB) [14]. Thus displaying such behavior on the part of the employees can only stem from their trust in their supervisors and chain of command [15]. High levels of trust in an organization result in fewer evaluation costs and provides the employees with more control and internal motivation [16]. A nurse manager’s role is also crucial with regards to the staff morale and trust as stated in a study by Wilson in which it is suggested that effective management can not only increase the work quality but also instill a sense of organizational commitment and grow their confidence in the nursing management [17].

Gaining the trust of nurses is important to ensure that effective and safe care is provided [18]. A treatment staff’s capability as a single mechanism is based on trust which enables them to enhance the work effectiveness and minimize errors and dissatisfactions. Thus, it is necessary now more than ever to give special attention to the matter of the trust between the managers and the nursing team in therapeutic environments [19]. Although numerous studies have been done about the importance of relations in the health care systems, less attention has been paid to inter-professional relations between nurses and managers [4, 15, 18, 20, 21]. Since employees’ trust in their managers as a key variable has a significant impact on their performance, so the present study was conducted to determine nurses’ trust in nursing managers, including head-nurses (the nurse in charge of the ward) and supervisors (the nurse who provide a link between hospital management and clinical care, overseeing patient-care operations, assigning and monitoring staff nurses and identifying and implementing quality improvements) and its relationship with nurses’ performance behavior.

## Methods

This study was descriptive- correlational research that was carried out in the year 2019. The study population included the entire nursing staff working in hospitals of the Ardabil University of Medical Sciences (*N* = 1126). The sample size was estimated to be 392 through Cochran’s sample size formula, which was calculated to be 431 with a 10% drop (t = 1.96, d = 0.04, *p* = 0.5).

$$ n=\frac{Nt^2 pq}{Nd^2+{t}^2 pq} $$

A stratified random sampling method was used for sampling so that the research community was divided into 5 classes based on therapeutic centers, and samples were selected from each class according to the sample size. Inclusion criteria included having a bachelor’s degree or higher in nursing, nursing work experience of more than 3 months, and interest in participating in the study. The nurses who had managerial positions (head- nurses, supervisors, nursing services managers) or those who did not work with patients were excludedfrom the study.

The data collection tool was a three-part questionnaire. Part 1 contained demographic characteristics (age, gender, ethnicity, marital status, education, work experience, managerial experience, employment status, work shifts, and workplace), Part 2; McAllister’s trust instrument (1995), and Part 3; Paterson’s job performance questionnaire (1922).

McAllister’s questionnaire comprised trust scale of 11 questions. The first six questions made up the cognition-based subscale and the following five questions made up the affect-based subscale. is rated on a 5-point Likert scale from 1 (strongly disagree) to 5 (strongly agree) and the overall scores fall into three categories;a low level of trust (12–14), a medium (25–36), and a high (37–60). The reliability and the validity of the questionnaire were also primarily measured in Cronbach’s alpha in a study that was conducted by Arizi, et al. (2012) which had yielded between 85 to 89% [22].

Paterson’s job performance consisted of 15 descriptive items that scored on a four-point Likert scale ranging from 1 (seldom) to 4 (always). The scores between1–15 were for poor performance, between 16 and 30 for moderate performance, 31–45 for good performance, and between 46 and 60 for very good performance. The validity and reliability of the questionnaire have already been evaluated and confirmed in the Feyzi study with Cronbach’s alpha coefficient of 0.90 [23]. In the present study, the Content Validity Index (CVI) was used to assess the validity of the questionnaires and was approved by 10 members of the Faculty of Ardabil School of Nursing and Midwifery. The CVI for the trust questionnaire was 93% and for the job performance questionnaire 98%. The data collection tool reliability was also assessed using Cronbach’s alpha coefficient which yielded 93% for the trust questionnaire and 90% for the job performance questionnaire.

After obtaining an official license from the Ardabil University of Medical Sciences and coordinating with the head of the hospital, security guards, and ward nurses, the researcher introduced himself to the research samples and after explaining the purpose of the research, obtained their written consent to participate in the research. All research samples were assured that the information obtained was confidential and did not need to be named.431 questionnaires were distributed, of which 417 handed over the questionnaires, and out of this number, 10 questionnaires were incompletely completed and were excluded from the study. Finally, data analysis was performed on 407 questionnaires (return rate of questionnaires = 94%).

Data analysis was carried out in SPSS software version 22. To analyze the data collected from descriptive statistics such as, mean, standard deviation, frequency distribution tables, graphs, and inferential statistics to determine the relationship between demographic characteristics (age, gender, ethnicity, marital status, level of education, work experience), Management history, employment status, work shift, hospital, and workplace ward) with the variables of trust, and behavioral performance, due to abnormal distribution of data by Kolmogorov- Smirnov test, Chi-square test was used. The significance level in statistical tests is considered 0.05.

## Results

An overall of 343 (84.3%) of the participants were women and 295 (72.5%) were married. A majority of the nurses were scheduled in the shift work mode (89.7%). The mean age was at 31/79 ± 6/14 years.

Regarding the status of nurses’ trust in managers, the results showed that 334 (82.1%) of nurses reported their trust in their head-nurses at a high level. Also, the status of nurses’ trust in the head- nurses in both affective and cognitive dimensions were reported high (Table 1).

**Table 1:** Frequency distribution of nurse trust in head-nurse and its dimensions

Trust	Low Number (%)	Medium Number (%)	High Number (%)
Affect-based Trust	25 (6.1)	76 (18.7)	306 (75.2)
Cognition-based Trust	12 (2.9)	67 (16.5)	328 (80.6)
Overall Trust	8 (2)	72 (17.7)	327 (80.3)

Regarding the nurse’s trust in the supervisor, the results showed that 252 (61.9%) of the nurses had high trust in the supervisor. Nurses’ trust in supervisors was also reported to be high in both affective and cognitive dimensions (Table 2).

**Table 2:** Frequency distribution of nurse-supervisor trust and its dimensions

Trust	Low Number (%)	Medium Number (%)	High Number (%)
Affect-based Trust	26 (6.4)	149 (36.6)	232 (57)
Cognition-basedTrust	25 (6.1)	129 (31.7)	253 (62.2)
Overall Trust	32 (7.9)	123 (30.2)	252 (61.9)

In terms of the functional behavior assessment of the nurses, the results revealed that a total of 377 of the nurses (92.6%) scored excellently and 30 nurses (7.4%) scored well for their functional behavior. No results were reported for average or poor performance (Table 3).

**Table 3:** Frequency distribution for the nurses’ functional behavior

Variable	Poor Number (%)	Average Number (%)	Good Number (%)	Excellent Number (%)
Functional Behavior	0	0	30 (7.4)	377 (92.6)

Regarding the relationship between nurses’ trust in the head-nurses and nurses’ functional behavior, the results of the chi-square test showed that there was no statistically significant relationship between the two variables (*P* = 0.580) (Table 4); While there was a significant relationship between nurses’ functional behavior and nurses’ trust in supervisors (*P* = 0.037) (Table 5).

**Table 4:** Relation between nurse trust in head-nurse and the nurses’ functional behavior

Variables	Variation	Functional Behavior		Statistical Test Results
		Good	Excellent	
		Number (%)	Number (%)	
Affective Aspect	Poor	2 (8)	23 (92)	χ^2^ = 0.507 *P* = 0.776
	Average	7 (9.2)	69 (90.8)	
	High	21 (6.9)	285 (93.1)	
	Total	30 (7.4)	377 (92.6)	
Cognitive Aspect	Poor	1 (8.3)	11 (91.7)	χ^2^ = 2.018 *P* = 0.991
	Average	5 (7.5)	62 (92.5)	
	High	24 (7.3)	304 (92.7)	
	Total	30 (7.4)	377 (92.6)	
Overall Trust	Poor	1 (12.5)	7 (87.5)	χ^2^ = 1.089 P = 0.580
	Average	7 (9.7)	65 (90.3)	
	High	22 (6.7)	305 (93.3)	
	Total	30 (7.4)	377 (92.6)

**Table 5:** Relation between nurse-supervisor trust and the nurses’ functional behavior

Variables	Variation	Functional Behavior		Statistical Test Results
		Good	Excellent	
		Number (%)	Number (%)	
Affective Aspect	Poor	3 (11.5)	23 (88.5)	χ^2^ = 5.485 *P* = 0.064
	Average	16 (10.7)	133 (89.3)	
	High	11 (4.7	221 (95.3)	
	Total	30 (7.4)	377 (92.6)	
Cognitive Aspect	Poor	3 (12)	22 (88)	χ^2^ = 4.922 *P* = 0.085
	Average	14 (10.9)	115 (89.1)	
	High	13 (5.1)	240 (94.9)	
	Total	30 (7.4)	377 (92.6)	
Overall Trust	Poor	2 (9.5)	19 (90.5)	χ^2^ = 6.606 P = 0.037
	Average	15 (12.2)	108 (87.8)	
	High	13 (4.9)	250 (95.1)	
	Total	30 (7.4)	377 (92.6)	

## Discussion

The present study was aimed to determine the nurses’ trust in managers and its association with the nurses’ functional behavior. The results showed the high trust of nurses in nursing managers. The results of different studies have reported different statuses of nurses’ trust in the organization. For instance, in the study by Ghanbari and Shemshadi, a similarly high level of organizational trust status among the nursing staff of the training centers of Hamedan was witnessed [4]. This is while SeyedJavadin (2014) and Farhang, et al. (2014) reported an average level of organizational trust in their reports; and Kafashpour, et al. (2012) also report a low level of organizational trust [15, 20, 24]. In another study conducted by Basit, et al. (2017) in three public hospitals in Antalya, Turkey suggested a low level of trust in the workplace organization; whereas the interpersonal trust among the nurses was high [18]. The above results can be due to differences in sampling method (available), tools used, and differences in the culture of the study population. What is important is that when the employees of a system trust their managers, they listen to their orders more willingly and pay more attention to the implementation of their activities to achieve the goals of the organization [25]. Managers and leaders can develop an atmosphere of trust in the organization by showing honesty and truthfulness and encouraging employees to do so, as well as the competencies and capabilities of leaders and managers and their belief in the importance of competence in doing things to increase and improve the level of trust in staff and organization. Adherence and fulfillment of managers and leaders to their commitments and promises will enable them to enjoy the necessary credibility in the organization and gain the trust of employees. In addition, transparency in the daily affairs of the organization, the behavior of managers, as well as the expression of goals and programs of the organization to employees strengthens the atmosphere of trust in the organization [26].

In the present study, the functional behavior of nurses was reported at an excellent level. In numerous other domestic and foreign studies in which the scoring was based on self-assessment, the job performance of the nurses was also reported as excellent [23, 27–29]. The results of Karim Yarjahromi’s (2013) study showed that the majority of nurses reported their job performance as desirable. So that nurses expressed the best performance in the areas of coordination and care activity and the lowest performance in the areas of clinical research and response to conflict [30]. The study of Khalifazadeh et al. (2012) also reported the quality of nurses’ performance as desirable [31]. In the study of Zamanzadeh et al. (2004), the findings showed that 42% of patients rated the quality of nursing care in the physical dimension, 32% in the psychosocial dimension, and 61% in the communication dimension, while these scores from the perspective of nurses were 92.6, 78.5 and 91.1%, respectively [32]. In the study of Neyshabouri et al. (2010), the quality of care in the psychosocial dimension was considered desirable from the perspective of 31.6% of patients and 92.6% of nurses, and in the communication dimension from the perspective of 24.7% of patients and 56.8% of nurses [33]. Shannon et al. (2002), who examined the different views of patients, nurses, and physicians on the quality of nursing care in intensive care units, reported the quality of care from the perspective of nurses at a good level [34]. However, in the study of Mosaferchi et al. (2018), the job performance status of nearly half of the nurses studied was less than acceptable, which could be due to the evaluation of nurses’ job performance by head- nurses [35]. While in the above studies, which examined the performance of nurses through self-assessment, the staff tends to report their performance more appropriately. Due to the weakness of self-assessment methods, it is recommended to check the performance of nurses in a checklist method to achieve more accurate results.

The findings of this study showed that there was a significant relationship between nurses’ functional behavior and the nurse’s trust in the supervisor. The study of Farajian (2013) showed a significant relationship between employees’ perceived trust in managers and its dimensions with staff performance [36]. Also, internal and external studies showed that both types of emotion-based and cognitive-based trust have a positive effect on organizational performance [25, 37]. On the other hand, the results of Ardam and Ozen (2003) study, which was conducted in a sample of 148 members from 28 teams in 4 organizations, showed that although there is a relationship between trust in the manager and organizational performance, but there are other factors that affect the need for more research in this area [38]. It seems that leaders and managers who have built and maintained employee trust have achieved more success when compared with those who did not. Trust in the manager affects components such as organizational citizenship behaviors and job performance [37]. Dirks (2000) showed that trust in the manager has a positive effect on organizational performance [39]. On the one hand, when employees trust their manager, they feel that the manager has paid attention to them, and this develops emotional-based trust and makes employees more willing to socialize and spend time [40]. In other words, a high level of affect-based trust builds better social relations between the managers and the members of the organization or the work unit in such a manner that enables the employees to become more responsive to their manager. On the other hand, the employees’ belief in the manager’s sense of responsibility, integrity, and competency can bring about the growth of cognition-based trust among them. Such trust motivated the employees to utilize more resources doing valuable work which ultimately results in organizational improved performance [41].

### Limitations

One of the limitations of this study was the multiplicity of supervisors in the centers, which made it difficult for nurses to decide to answer the questions. The other limitation was personality differences, attitudes, and perceptions of nurses towards interaction and trust with nursing managers that could affect their expression. The use of self-report tools also leads to bias and more or less estimation of consequences. The lack of similar research, especially in the country to compare the results is another limitation of this study. The optimal responsebias, which is the tendency of some people to report desirable individual characteristics and less reporting undesirable social characteristics, was another limitation that was beyond the control of the researcher.

## Conclusion

This study showed that the majority of nurses in both affective and cognitive dimensions of trust had a high score indicating high trust of nurses in nursing managers including head-nurse and supervisor. In addition, nurses’ functional behavior was reported to be excellent. The results showed a statistically significant relationship between nurse-supervisor trust and nurses’ functional behavior. Understanding the nurses’ trust in managers can help health care providers plan for achieving better engagement of nursing staff with nursing managers, which are important issues in improving the quality of patient care and safety. In this regard, hospital managers should pay attention to the views and opinions of nurses and apply them as far as practicable and strive to create a positive vision and feeling for nurses in line with current hospital goals and activities.

## Data Availability

The datasets used and/or analyzed during the current study are availablefrom the corresponding author on reasonable request.

## Abbreviations

MSc: Master of Science
OCB: Organizational Citizenship Behaviors
Ph.D: Doctor of Philosophy
